# The type-2 *Streptococcus canis* M protein SCM-2 binds fibrinogen and facilitates antiphagocytic properties

**DOI:** 10.3389/fmicb.2023.1228472

**Published:** 2023-10-26

**Authors:** Antje-Maria Lapschies, Etienne Aubry, Thomas P. Kohler, Oliver Goldmann, Sven Hammerschmidt, Andreas Nerlich, Inga Eichhorn, Kira van Vorst, Marcus Fulde

**Affiliations:** ^1^Centre of Infection Medicine, Institute of Microbiology and Epizootics, Freie Universität Berlin, Berlin, Germany; ^2^Centre for Functional Genomics of Microbes, Department of Molecular Genetics and Infection Biology, Interfaculty Institute for Genetics and Functional Genomics, University of Greifswald, Greifswald, Germany; ^3^Infection Immunology Group, Helmholtz Centre for Infection Research, Braunschweig, Germany; ^4^Veterinary Centre for Resistance Research (TZR), Freie Universität Berlin, Berlin, Germany; ^5^Robert Koch Institute, Genome Competence Centre (MF1), Berlin, Germany

**Keywords:** *Streptococcus canis*, SCM, fibrinogen-binding, anti-phagocytosis, pathogen-host interaction

## Abstract

*Streptococcus canis* is a zoonotic agent that causes severe invasive diseases in domestic animals and humans, but little is known about its pathogenesis and virulence mechanisms so far. SCM, the M-like protein expressed by *S. canis*, is considered one of the major virulence determinants. Here, we report on the two distinct groups of SCM. SCM-1 proteins were already described to interact with its ligands IgG and plasminogen as well as with itself and confer antiphagocytic capability of SCM-1 expressing bacterial isolates. In contrast, the function of SCM-2 type remained unclear to date. Using whole-genome sequencing and subsequent bioinformatics, FACS analysis, fluorescence microscopy and surface plasmon resonance spectrometry, we demonstrate that, although different in amino acid sequence, a selection of diverse SCM-2-type *S. canis* isolates, phylogenetically representing the full breadth of SCM-2 sequences, were able to bind fibrinogen. Using targeted mutagenesis of an SCM-2 isolate, we further demonstrated that this strain was significantly less able to survive in canine blood. With respect to similar studies showing a correlation between fibrinogen binding and survival in whole blood, we hypothesize that SCM-2 has an important contribution to the pathogenesis of *S. canis* in the host.

## Introduction

The group G beta-hemolytic *Streptococcus canis* commonly colonizes the skin and mucosal surfaces of dogs and cats. Furthermore, *S. canis* was also identified in various other animal hosts, such as cows, rats, minks, mice, rabbits and foxes (Corning et al., 1991; Iglauer et al., 1991; Chaffer et al., 2005; Verkühlen et al., 2016; Nikolaisen et al., 2017; Timoney et al., 2017). As an opportunistic pathogen, *S. canis* infections generally lead to local and self-limiting alterations of skin and mucosa, but in some cases it can proceed to severe and life-threatening diseases, such as streptococcal toxic shock-like syndrome (STSLS), necrotizing fasciitis (NF), meningitis and septicemia (Miller et al., 1996; DeWinter et al., 1999; DeWinter and Prescott, 1999; Lamm et al., 2010). There is evidence that *S. canis* displays some zoonotic potential, since it can spread among several host species (Galpérine et al., 2007; Lam et al., 2007; Fulde and Valentin-Weigand, 2013; Ohtaki et al., 2013; Lacave et al., 2016; Taniyama et al., 2017). Genetic analysis has shown that the closest relatives to *S. canis* are *Streptococcus dysgalactiae* and *Streptococcus pyogenes* (the former being a growing human pathogen and the latter being a prominent one) (Brennan-Krohn, 2021). The zoonotic capacity of *S. canis* coupled with its potential to cause life-threatening diseases renders it a worrying prospect as an emerging pathogen.

Little is known of the pathogenesis of *S. canis* to date. The most relevant finding was the identification of an M-like protein comparable to that shown by Rebecca Lancefield in *S. pyogenes* (group A streptococci, GAS) (Robinson and Kehoe, 1992). The M protein of *S. pyogenes* is a comprehensively studied virulence factor which is currently used as the main tool in pathogen classification. There are over 200 different groups of the M protein in GAS alone with a high variability in the function of the M protein, leading to different pathogenic proceedings. For example, plasminogen binding M proteins encourage dissemination of streptococci due to the activation of the bound plasminogen to plasmin by host-derived factors such as urokinase. Fibrinogen binding serves in immune evasion by initiation of opsonophagocytosis via masking complement protein C3b (Laabei and Ermert, 2019).

The versatility and importance of the GAS M protein for pathogenesis is indisputable. Given the close phylogeny of *S. pyogenes* and *S. canis*, further studies on *S. canis* M-like protein (SCM) for similar pathogenic trends are urgent. SCM was discovered more than 10 years ago (Yang et al., 2010; Fulde et al., 2011). At that time, it was asserted that not all strains of *S. canis* were capable of expressing SCM. The M protein was shown to have high plasminogen and immunoglobulin G (IgG) binding capabilities, comparable to functions shown for GAS M proteins (Fulde et al., 2013; Bergmann et al., 2017; Nerlich et al., 2019). Binding might facilitate conversion of bound plasminogen to plasmin and thereby promote host dispersion by degrading extracellular matrix molecules and dissolving aggregated fibrin thrombi in *S. canis* (Fulde et al., 2011). Alternatively, plasminogen binding has been shown to mediate antiphagocytic activity. Binding capacity of plasminogen by SCM alongside enolase increased SCM reassociation, which then led to aggregation of *S. canis*. This aggregation could impede phagocytosis and is assumed to be one part of the antiphagocytic activity of *S. canis* (Fulde et al., 2013). IgG binding by *S. canis* had a similar effect: *S. canis* bacterial cells aggregated in the presence of IgG, but this IgG-derived aggregation is only feasible in SCM-expressing *S. canis* (Nerlich et al., 2019). Furthermore, binding of IgG is presumed to prevent opsonization by C1q, conferring another antiphagocytic property (Bergmann et al., 2017). Interestingly, a previous study indicated that *S. canis* is also capable of binding human fibrinogen like GAS, although the mechanisms behind this phenomenon have to be elucidated (Lämmler et al., 1988). More recently, a study by Pinho et al. (2019) discovered that the SCM protein is in fact universally present in all *S. canis* strains and that previously identified *scm* negative *S. canis* strains were actually proven to possess a genetically divergent form of the SCM protein. This laid the groundwork to assert that the SCM protein can be divided into two groups: SCM type 1 (SCM-1), which is capable of binding plasminogen and IgG and SCM type 2 (SCM-2), whose function has yet to be determined.

In this study, we demonstrate that the fibrinogen binding capability of *S. canis* is linked to the presence of SCM-2 via FACS and surface plasmon resonance spectrometry (SPR) analysis.

## Materials and methods

### Bacterial strains and growth conditions

The *scm2* positive *S. canis* strains IMT40096 and IMT42870 were isolated by routine diagnostics at Freie Universität Berlin in 2016, as well as *scm1* positive *S. canis* strain IMT40165. Bacteria were routinely grown in Brain Heart Infusion (BHI) or on Brain Heart Agar (BHA) at 37°C without shaking. In preparation for electroporation, IMT40096 was grown overnight in BHI and subcultured into 50 mL of BHI the following day. The resulting culture was harvested at an OD of 0.5 at a wavelength of 600 nm by centrifugation (10 min at 3250 × *g*), washed three times in 50 mL 20% sterile glycerol solution, and resuspended in 1 mL of 10% glycerol solution. Aliquots of 100 μL were kept frozen at −80°C until needed. *Escherichia coli* (*E. coli*) EC101 were cultivated in Luria-Bertani media (LB). For chemical competence, EC101 was subcultured into 50 mL of LB and grown at 37°C 200 rpm until an OD of 0.6 at 600 nm was reached. The resulting suspension was put on ice for 1 h, washed twice with 10 mL of 0.1 M CaCl_2_ by centrifugation at 7000 rpm for 7 min, put on ice for 1 h to be then resuspended in 1 mL of 0.1 M CaCl_2_. Aliquots of 100 μL were produced with 10% glycerol and stored at −80°C until necessary. Where indicated, antibiotics were supplemented as follows. *E. coli*: erythromycin (Erm) 200 μg/mL; *S. canis*: Erm 2 μg/mL.

### Whole-genome sequencing by Illumina

DNA extraction was conducted with the QIAmp DNA kit according to manufacturer’s instructions. DNA measurements were made with the Qubit^™^ 2.0 Fluorometer with the dsDNA HS.

The libraries for whole-genome sequencing (WGS) were prepared using the Nextera XT DNA Library Preparation Kit (Illumina, Inc., San Diego, United States) according to the manufacturer’s recommendations. The 2 × 300 bp paired-end sequencing in 40-fold multiplexes was performed on the Illumina MiSeq platform (Illumina, Inc., San Diego, United States) with MiSeq Reagent Kit v3 (600 cycle).

### Sequence assembly

The raw Illumina reads were quality checked by FastQC (Andrews, 2010) and trimmed by Trim Galore v0.6.6 (RRID:SCR_011847). *De novo* assembly into contigs was carried out with Unicycler (Wick et al., 2017).

### Protein alignment and tree construction

Protein alignment was conducted with the ClustalW webtool (Minh et al., 2020). A Maximum Likelihood tree was then constructed with the IQtree v2.2.0.3 software while performing 1,000 boostraps with UFBoot2 and the best fit model selected by ModelFinder (Sievers et al., 2011; Kalyaanamoorthy et al., 2017; Hoang et al., 2018). Tree visualization was performed with the iTOL: Interactive Tree of Life webtool (Letunic and Bork, 2021).

### Antibodies and reagents

Human FITC-labeled fibrinogen was purchased from Sigma. Ethidium homodimer-1 and the secondary rabbit anti-mouse IgG Alexa Fluor 488-conjugated antibody (Cat#: A-11059; RRID: AB_2534106) were obtained from Life Technologies. 16% formaldehyde without methanol and CitiFluor^™^ CFM3 mounting medium were obtained from Electron Microscopy Science.

### Construction of the IMT40096 *scm2* targeted insertional mutant

Targeted insertional mutagenesis of *scm2* was performed using the pGh9:Δ*scm* vector (Maguin et al., 1992) obtained by inverse PCR of the pGh9:ISS1 vector (NCBI: txid481005) with the following primer pair which included *NcoI* restriction sites: 5′-GGGCCATGGGCTCCTTGGAAGCTGTCAG-3′; 5′-CCCCCATGGGGTACCCAATTCGCCCTATA-3′. The vector was then inserted into *E. coli* EC101 via chemical competence and grown for 2 days in erythromycin to induce plasmid replication. The *scm2* gene with 500 bp upstream and downstream of it was amplified by PCR using the following primer pair which also included *NcoI* restriction sites: 5′-GGGCCATGGGACATCGTGAATTTTGCCG-3′; 5′-CCCCCATGGCACTTGGATAGGATGCAC-3′. PCR products were cloned into the temperature-sensitive vector pGh9: ΔISS1 by *Nco*I digestion and alkaline phosphatase to avoid religation products. The resultant plasmid was then amplified by inverse PCR to knock out *scm2* while keeping the 500 bp regions around it. This was done with the following primer pair which included *EcoRV* restriction sites at the 500 bp regions around *scm2*: 3′-GGGGATATCGTGACAGGCTATCTTTAG-5′; 3′-CCCGATATCAGCCTCGTTACAGACTATCC-5′. The plasmid was then inserted into EC101 to produce pGh9:Δ*scm2* which was verified by band size measurement after *EcoRV* and *NcoI* digestion (1 band at 3500 bp representing the vector and 2 bands at 500 bp representing the 500 bp regions around *scm2*). One μg of the resultant knockout plasmid was introduced into IMT strain 40096 by electroporation, and Erm resistant transformants were identified at the permissive temperature for plasmid replication (28°C). Single-crossover Campbell-type chromosomal insertion was selected by shifting to the nonpermissive temperature (37°C) while maintaining Erm selection. SCM phenotype was determined on BHA plus Erm at 37°C. Verification of the mutagenesis was conducted via PCR using the *scm2* amplification primers described above.

### Expression and purification of recombinant SCM-2

Protein extraction was performed as described previously (Nerlich et al., 2019). Briefly, *scm* fragments from different *S. canis* strains were overexpressed in *E. coli* via Qiagen’s pQE30 expression vector, followed by purification of the recombinant histidine-tagged proteins as described by the manufacturer (Qiagen ExpressionistTM System).

### Whole blood assays

Survival of *S. canis* wild-type and the isogenic *scm2* mutant in blood was analyzed using whole heparinized canine blood. Handling of dogs and sampling was conducted in strict accordance with the principles of the European Convention for the Protection of Vertebrate Animals Used for Experimental and Other Scientific Purposes as well as the German Animal Protection Law. Blood was retrieved due to excess in diagnostics procedures.

Briefly, 1 mL canine whole blood was mixed with 1 × 10^5^ CFU of wild-type or mutants. The mixture was incubated on a rotator at 37°C for up to 6 h with sampling each hour. Survival factors were determined by dividing the number of CFU after any incubation period at 37°C by the number of CFU at *t* = 0 min (CFU were determined by plating of serial dilutions on BHI plates). Three blood samples from different dogs were used.

### Immunofluorescence staining and confocal microscopy

Immunofluorescence staining and confocal microscopy was performed as described previously (Nerlich et al., 2019). In brief, 100 μL of bacterial overnight culture was allowed to adhere to poly-L-lysinecoated μ-slide 8-well (ibidi) filled with 100 μL PBS for 30 min and subsequently fixed with 3% PFA for 15 min. After washing with PBS samples were incubated with rabbit anti-mouse Alexa Fluor 488-conjugated IgGs in PBS/1% BSA/Tween 20 overnight at 4°C and bacterial RNA/DNA was stained with ethidium homodimer-1. Samples were mounted in CitiFluor^™^ CFM3 and examined using an LSM 780 confocal laser-scanning microscope with a PlanApochromat 63×/1.4 NA objective driven by Zen 2012 software (Carl Zeiss). Image stacks with a z-step size of 0.2 μm per plane were acquired with the pinhole set to 1 airy unit in sequential imaging mode to avoid bleed-through of fluorescence emission. Images were deconvolved using Huygens^®^ Essential 16.10 (Scientific Volume Imaging) and 3D-stacks are displayed as maximum intensity projections and adjusted identically for brightness and contrast in ImageJ/Fiji (Schindelin et al., 2012).

### Flow cytometric analyzes of IgG binding and fibrinogen binding (FACS)

*S. canis* IMT40096 and IMT40096Δ*scm*2 were grown to mid-exponential phase in TSB medium and washed once in PBS. Bacteria are adjusted to a transmission of 10% equal to 10^8^ bacteria per mL. A total of 1 × 10^7^ bacteria were suspended in 400 μL of PBS containing 0.5% FCS and incubated with 0.5 mg polyclonal rabbit IgG for 30 min at 37°C. After washing in PBS the bacterial pellet was suspended in 100 μL of PBS containing an anti-rabbit ALEXA^®^ Fluor 488 antibody or FITC-labeled fibrinogen, respectively as described previously and incubated for 30 min at 37°C. Bacteria were washed again in PBS, fixed in PBS containing 3% paraformaldehyde and analyzed by flow cytometry using a FACS LSRII (Becton Dickinson). Cells were analyzed with the FlowJo software v.7.6.5. Cells were gated on Side scatter area (SSC-H) vs. Fluorescin isothiocyanate labeled human fibrinogen from Molecular Innovations (FITC-H). IMT40096 was determined as the positive control and used as the gating reference for the succeeding samples (Supplementary Figure S1). The gating region was set to exclude debris and larger aggregates of bacteria. A total of 10^4^ bacteria were analyzed for fluorescence using log-scale 2 amplification.

### Surface plasmon resonance spectrometry

The interaction of SCM-1 or SCM-2 with fibrinogen was analyzed by surface plasmon resonance spectroscopy using a Biacore T200 optical biosensor (GE Healthcare). Human fibrinogen (Molecular Innovations) was immobilized (~1,000 response units) on a carboxymethyl dextran sensor chip (CM5) via amine coupling as described previously (Bergmann et al., 2017). SCM proteins were used as analytes in a concentration range of 1.25–20 μg/mL. Binding analysis was performed in PBS containing 0.05% Tween20 at 25°C and a flow rate of 10 μL/min. Data were analyzed using Biacore T200 evaluation software (version 3.2).

## Results

### Identification of two distinct SCM groups

**Figure 1:**
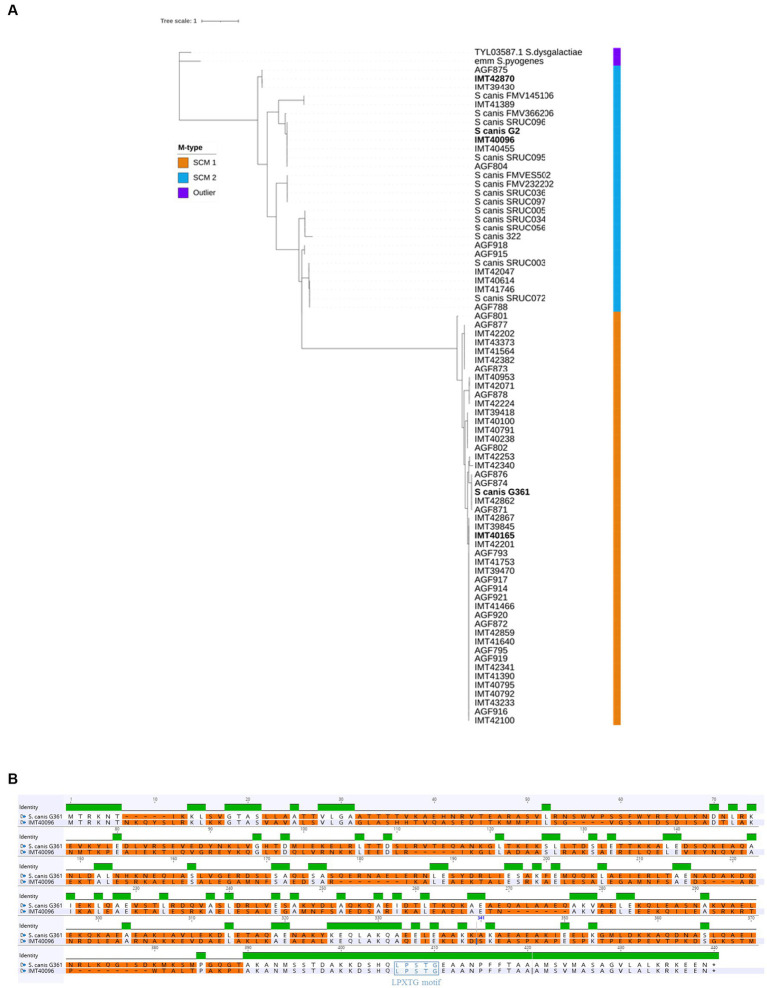
Comparing SCM-1 and SCM-2. **(A)** Alignment tree of SCM protein sequences of 75 clinical *S. canis* isolates from different host species, 14 reference strains and 2 outliers. Maximum Likelihood tree generated with IQtree and visualized on iTOL. Strain names in bold were used in further experiments. **(B)** Protein Sequence alignment of a representative SCM-1 and SCM-2 protein (*S.canis* G361 and IMT40096, respectively). Green represents regions with 100% sequence identity between SCM-1 and SCM-2. The absence of a colored bar indicates that no consensus was observable between SCM-1 and SCM-2 in that region. Differences between the amino acid sequences were highlighted in orange. The membrane anchoring LPXTG motif was highlighted in purple.

Alignment of the *scm* nucleotide sequences of 75 clinical isolates of *S. canis* (Supplementary Table 1) via Maximum Likelihood revealed the presence of two genetically distinct populations differentiated by the SCM protein (Figure 1A). *S. canis* strain G361 and IMT40165 used in this study belonged to the same population (hereby referred to as the SCM-1 strains), but had a significantly different *scm* gene compared to *S. canis* strains G2, IMT40096 and IMT42870, which formed their own population (SCM-2). Alignment of SCM-1 proteins to SCM-2 showed an identity as low as 35%. However, closer inspection of the sequences showed that the C-terminal region of the two proteins corresponding to the sortase-anchoring motif LPXTG motif is highly conserved (Figure 1B). Given these differences, we wondered if the IgG binding capabilities previously observed in SCM-1 protein expressing *S. canis* were conserved by the SCM-2 protein.

### SCM-2 *Streptococcus canis*, contrary to SCM-1, does not bind IgG

**Figure 2:**
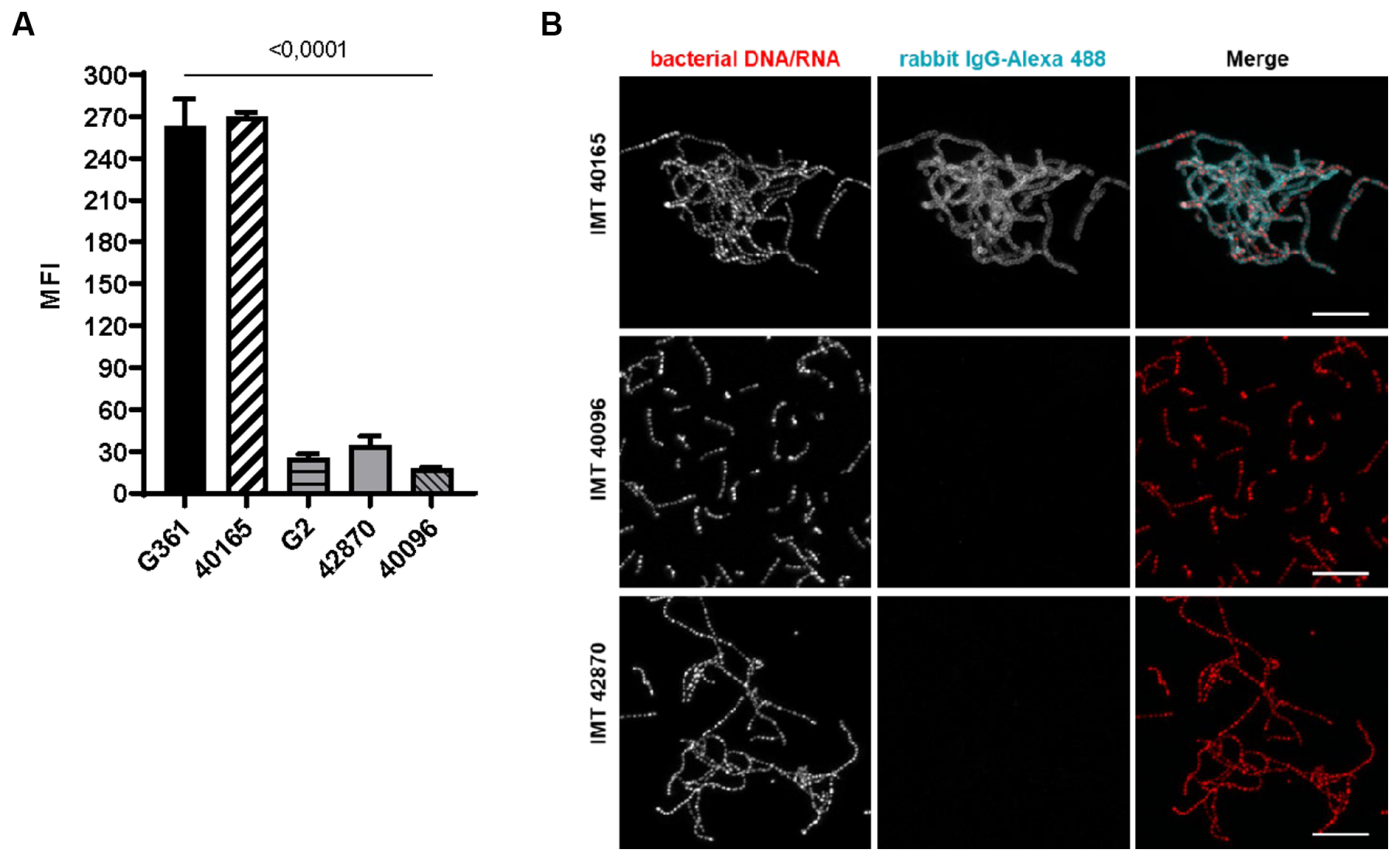
IgG binding of SCM-2 *S. canis* strains **(A)** FACS analysis with Alexa 488 conjugated. IgG Positive controls: SCM-1 *S. canis* G361 and IMT40165. Negative control: *S. canis* G2 (*n* = 3). Data represent mean fluorescence intensity (MFI) ± SD of three independent experiments. Statistical significance was calculated with ordinary one-way ANOVA. **(B)** Confocal microscopic analysis of bacterial aggregation and IgG binding. Strains were grown overnight at 37°C in TSB. Bacteria were allowed to adhere to poly-L-lysin-coated ibidi-slides, fixed and incubated with Alexa 488-conjugated rabbit IgG (cyan). Bacterial DNA/RNA was stained with ethidium homodimer-1 (red). Bacterial aggregation was visualized by confocal microscopy. Positive control: *S. canis* IMT40165. Bars indicating 10 μm.

In a previous study, we identified IgG binding as a function enabled by SCM-1 (Bergmann et al., 2017). Consequently, we investigated whether similar properties are conferred by SCM-2. FACS analysis of SCM-2 *S. canis* showed significantly reduced IgG binding when compared to G361 SCM-1 reference strain. When quantifying IgG binding, SCM-2 of G2, IMT40096 and IMT42870 displayed a 10-fold lower geometric mean fluorescence intensity (MFI) than G361 SCM-1 (Figure 2A). Correspondingly, SCM-2 strains do not appear to be as efficient in binding IgG.

In accordance with findings by Nerlich et al. (2019), fluorescence microscopy of IMT40165, IMT40096 and IMT42870 showed IgG binding properties and a bacterial aggregation only by *S. canis* strain IMT40165. In contrast, both SCM-2 strains were negative and did neither bind IgG nor aggregate in its presence (Figure 2B).

### SCM-2 expressing *Streptococcus canis* strains bind fibrinogen

**Figure 3:**
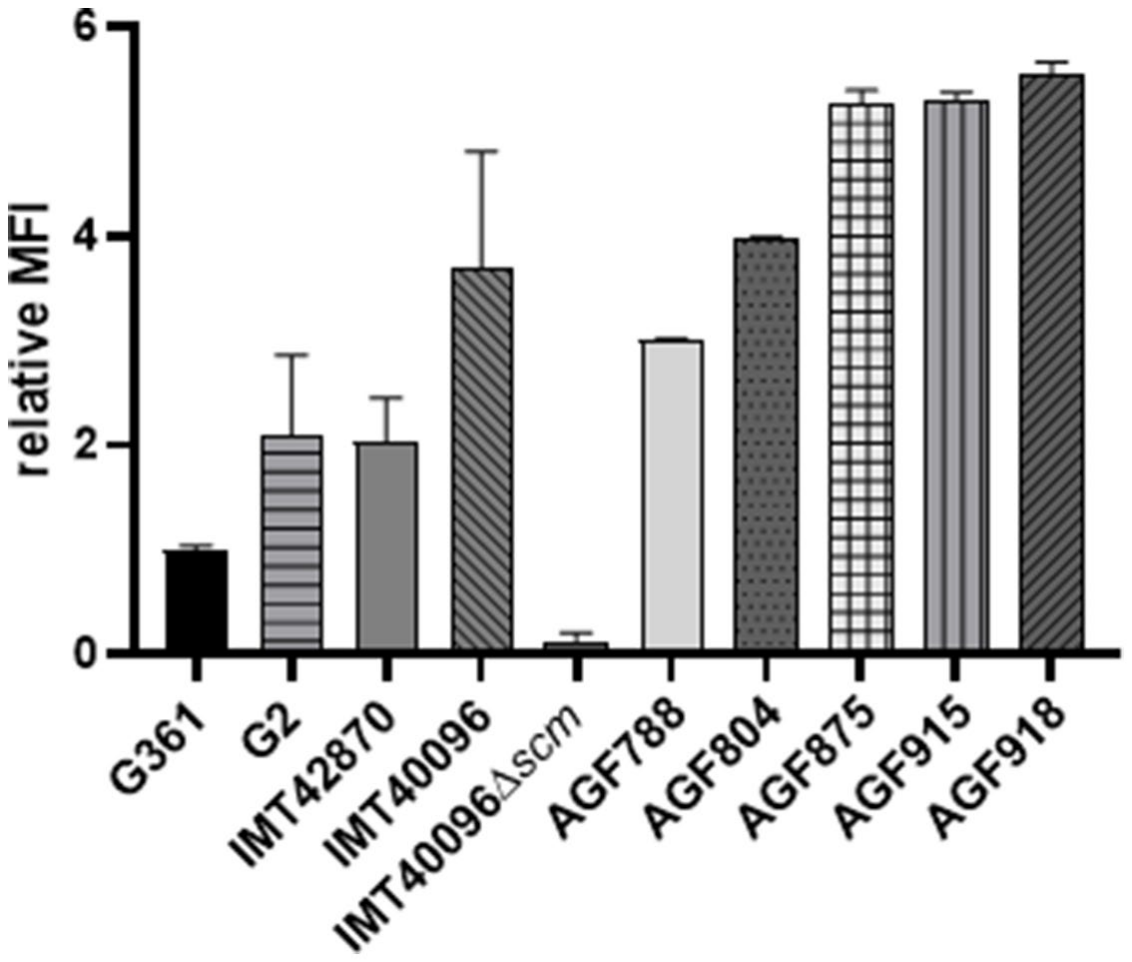
Analysis of the fibrinogen binding capacities of different SCM-2 expressing *S. canis* strains. FACS analysis with FITC-conjugated human fibrinogen. Positive control: *S. canis* G2. Negative control: *S. canis* G361. Data represent MFI ± SD of three independent experiments normalized to the fluorescence of SCM-1 *S. canis* G361. Fibrinogen binding is abrogated in the KO mutant IMT40096Δ*scm* (*n* = 4). Statistical significance was calculated with unpaired *t*-test.

Previous studies on M proteins belonging to *Streptococcus pyogenes* clade Y and *Streptococcus equi* ssp. *zooepidemicus* (SEZ) showed that certain M proteins are capable of binding fibrinogen (Sanderson-Smith et al., 2014; Bergmann et al., 2019). Hence, we assessed fibrinogen binding capacity of *S. canis* in the next step. FACS analysis of SCM-1 *S. canis* 361 versus SCM-2 *S. canis* revealed increased human fibrinogen binding in SCM-2 expressing strains. In comparison to the *scm1* expressing *S. canis* G361, the fluorescence of SCM-2 *S. canis* strain IMT40096 was more than three times higher. In fact, all other SCM-2 strains tested shared an increase in fluorescence corresponding to fibrinogen binding by at least two-fold (Figure 3). Furthermore, deletion of *scm2* in IMT40096 background diminished fluorescence to 0.13 compared to the control strain G361. These results suggest that the *scm* gene is at the center of fibrinogen binding in the tested SCM-2 *S. canis* strains (Figure 3).

**Figure 4:**
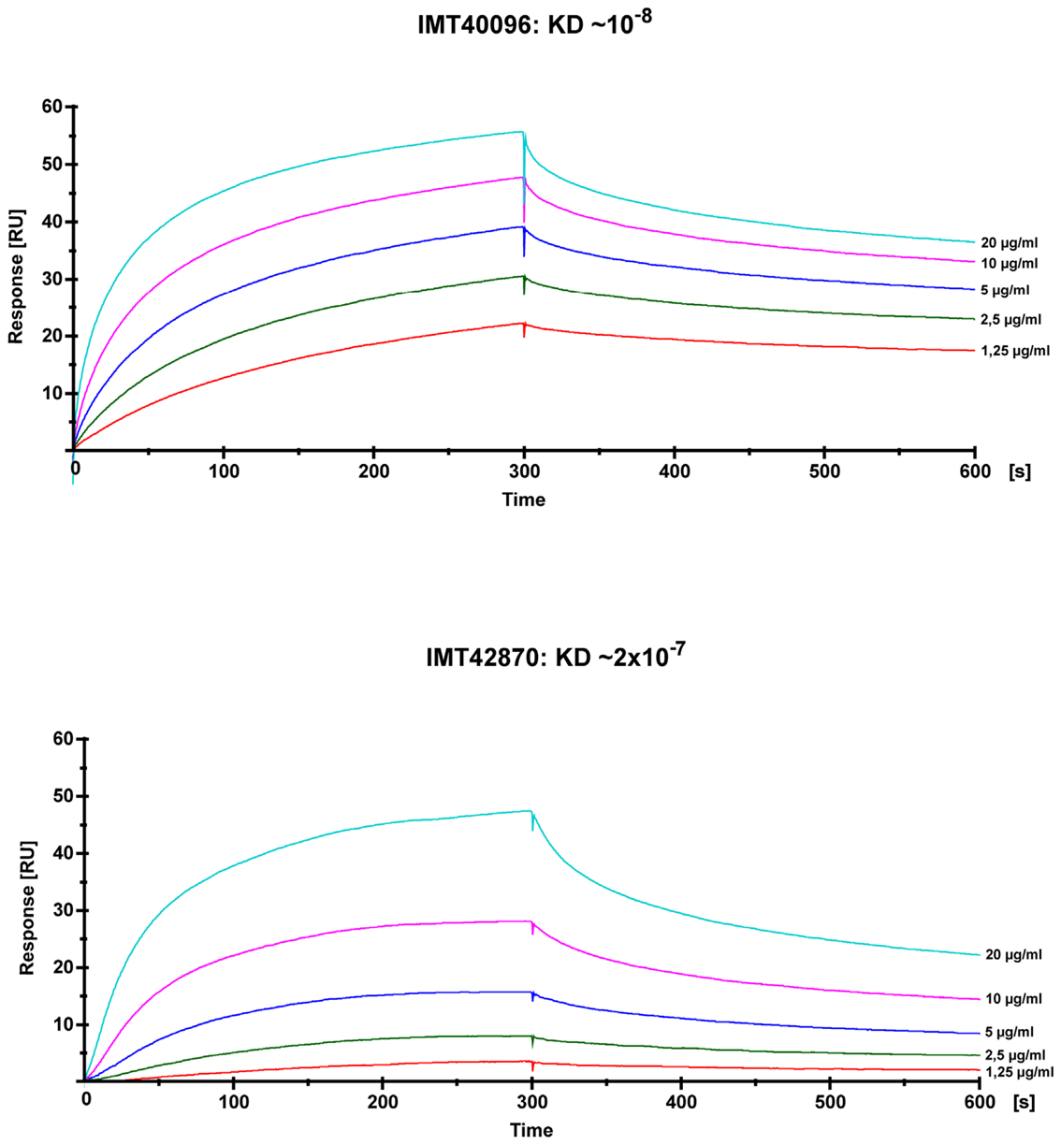
Analysis of the fibrinogen binding capacities of SCM-2 expressing *S. canis* strains IMT40096 and IMT42870. Interactions of soluble SCM with immobilized fibrinogen were analyzed by surface plasmon resonance spectroscopy (SPR). Representative sensorgrams of three independent experiments of two SCM-2 expressing *S. canis* (IMT40096, IMT42870). Histidine-tagged recombinant proteins were extracted as previously described (Fulde et al., 2013). The association and dissociation was observed, each of 300 s. Values of the control flow cells were subtracted from each sensorgram. A representative KD value was calculated for the interaction between fibrinogen and SCM using the 1:1 Langmuir binding model.

SPR analysis to evaluate the SCM protein’s affinity to fibrinogen showed KDs of around 10^−8^ for IMT40096 and 2 × 10^−7^ IMT42870, indicating a strong affinity for fibrinogen for the SCM proteins of both strains (Figure 4; Supplementary Figure S2). This is particularly remarkable bearing in mind that both SCM-2 strains originate from different branches of the Maximum Likelihood Tree (Figure 1) and hence display low structural similarities. Nevertheless, functional traits and fibrinogen affinity seems to be conserved. Contrary, fibrinogen binding was not observed for SCM-1 expressing *S. canis* strain G361 (Supplementary Figure S3). This demonstrates fibrinogen binding capabilities of SCM-2 at protein level.

### SCM-2 is crucial for the survival of *Streptococcus canis* in whole blood

**Figure 5:**
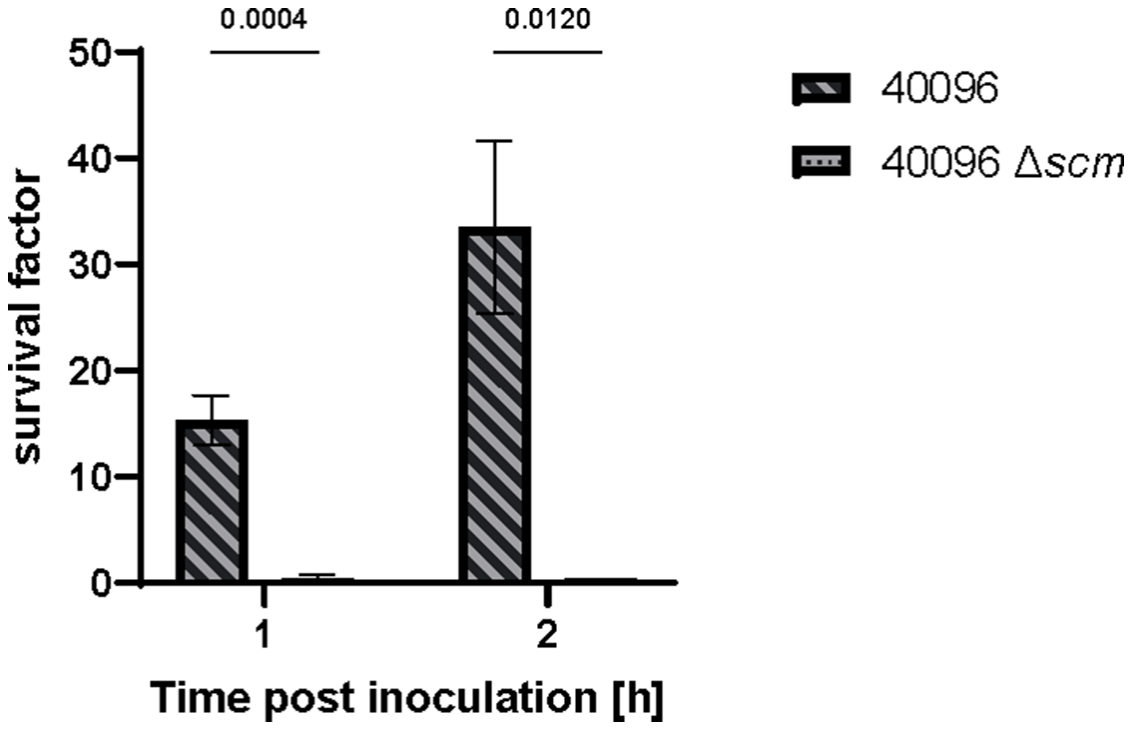
Whole blood assay of *S. canis* IMT40096 WT and IMT40096Δ*scm*. Canine blood was heparinized and incubated with 1 × 10^5^ CFU of *S. canis* for up to 2 h. Survival factors were calculated by ratio of CFU counts at timepoint 0 h versus 1 and 2 h post-inoculation (*n* = 3). Statistical significance was calculated with unpaired *t*-test.

Finally, we evaluated pathophysiological consequences of SCM-2 deletion in *S. canis* performing a functional assay. Therefore, survival of IMT40096 and its SCM mutant in canine whole blood was assessed. A significant decrease in survival was observed for the mutant strain compared to the wild-type (Figure 5). At 1 h post-inoculation, the wild-type showed a survival factor of approximately 4, whereas the SCM mutant displayed a survival factor of only 0.5. This difference was even more exacerbated when inoculation was extended to 2 h, where the wild-type reaches survival factors of up to 10 while the SCM mutant stagnates at 0.5. This suggests that, as shown for other M proteins in GAS and *S. dysgalactiae* spp. *equisimilis* (Whitnack and Beachey, 1985; Carlsson et al., 2005), SCM-2 and associated fibrinogen binding is critical for the survival of *S. canis* 2 in canine whole blood.

## Discussion

In this study, we extent knowledge on SCM-2 properties and confirm the organization of *S. canis* into two distinct SCM groups in accordance with previous statements of Pinho et al. (2019). IgG-binding of SCM-1 was first identified by Bergmann et al. (2017) and Pinho et al. (2019) later identified *S. canis* with and without this ability as SCM-1 and SCM-2, respectively. Here, we demonstrate that the protein encoded by *scm2* is necessary for SCM-2 streptococci to bind fibrinogen and that its presence correlates with the ability of type 2 *S. canis* IMT40096 to survive in whole blood; similar to what has been observed by Bergmann and colleagues in SEZ via SzM previously (Bergmann et al., 2019).

The cognition that *S. canis* is capable of binding fibrinogen corresponds with former findings for SEZ and *S. pyogenes* (Carlsson et al., 2005; Bergmann et al., 2019). SEZ was demonstrated to be important for resistance of opsonophagocytosis by reducing C3b deposition in *S. pyogenes*, which in turn inhibits the activation of the classic complement pathway. Furthermore, M protein-deficient *Streptococcus equi* had decreased survival in whole blood, most likely due to the loss of host-immune evasion conferred by the protein. Likewise, SCM-2 of *S. canis* strain IMT40096 has been shown to contribute to the survival of *S. canis* in canine whole blood in the present study, suggesting a potent role of SCM2 for pathogenesis. However, it should be noted at this point that, in contrast to the fibrinogen binding studies, the survival studies in canine blood shown in this manuscript refer only to one representative *S. canis* SCM-2-type isolate. A global statement that all SCM-2 proteins confer for survival in whole blood is therefore not possible. Further studies, e.g., the inclusion of complemented SCM-2 deficient strains or the use of (inhibitory) antibodies directed against SCM-2, should be part of future studies.

SCM-1 facilitates plasminogen recruitment, leading to increased bacterial survival in the presence of human blood neutrophils (Fulde et al., 2011, 2013). SCM-1 *S. canis* has been reported to bind mini-plasminogen due to the cooperation with surface enolase. This effect coupled with the capacity of SCM to self-aggregate significantly inhibits phagocytosis of *S. canis* and therefore allow it to persist in blood (Fulde et al., 2013). By comparison, we demonstrate that SCM-2 allows binding of fibrinogen, which confers a fitness advantage as *scm* deletion led to significantly reduced recovery after incubation in canine whole blood in the present study. In sum, it is therefore conceivable to distinguish two strategies of *S. canis* pathogenesis in terms of survival in blood enabled by the respective SCM type.

It remains unclear why the *scm* gene has two distinct groups. Both enable binding to host proteins and subsequent immune evasion. For SCM-1 strains of *S. canis*, this is due to the interaction between the SCM protein and the Fc domain of IgG on the bacterial surface, thereby preventing opsonisation by C1q (Bergmann et al., 2017). Furthermore, IgG provokes the aggregation of *S. canis* as well, which might facilitate avoidance of host-immunity by resisting phagocytosis (Nerlich et al., 2019). The mechanism behind host immune evasion by SCM-2 remains to be elucidated, but a parallel may be drawn to the fibrinogen binding properties observed in *S. pyogenes*. Binding of fibrinogen on the bacterial surface leads to inhibition of the classical pathway of complement via C3b. Streptococcal SCM-2 proteins may serve a similar function (Whitnack and Beachey, 1985).

Overall, the finding that all investigated SCM-2 *S. canis* bind fibrinogen is an important step in unravelling differences between SCM-1and SCM-2 strains and suggests that the two strains evolved under distinct conditions. However, since no host nor infection type-related classifications can be drawn, postulation of probable explanations for this group division process is compounded (Pinho et al., 2019). Studies conducted with *S. pyogenes* evidenced that *emm* typing can be clustered based on the function of the M protein. Following this principle, two *emm* clades are formed: clade X is primarily associated with *S. pyogenes* expressing M proteins capable of binding IgG, C4bP and albumin. Clade Y mainly binds fibrinogen and albumin, but some strains of this clade can bind plasminogen as well. It is interesting to note that the fibrinogen binding and IgG binding M proteins are in two different clades, similar to the divide between SCM-1 and -2 strains of *S. canis* (Smeesters and Botteaux, 2020). In future studies, we aim to test both SCM types for C4bP and albumin binding to see if a trend forms.

Studies by Fukushima et al. (2020) have shown a correlation between SCM type and antimicrobial resistance genes. SCM-2 *S. canis* have been shown to exhibit an increased likelihood of non-susceptibility to tetracycline and macrolide/lincosamide class antibiotics. The link between the two occurrences is unclear to date. It is possible that fibrinogen binding may play a role in tolerance against antibiotics, which could eventually lead to resistance.

Exposing the bacteria to canine and human macrophages to quantify survival could reveal if the binding of fibrinogen indeed influences opsonisation. Intraperitoneal infection of the bacteria into mice could demonstrate the overall impact of the *scm-2* gene on pathogenesis. Testing the SCM-2 strains of *S. canis* for C4BP binding could also be interesting due to the high prevalence of this function in *S. pyogenes*. These experiments could lead to a better understanding of the mechanisms behind the decreased survival of *S. canis* in whole blood after deletion of *scm-2*. Epidemiological surveys which specifically identify differing trends between *S. canis* strains of type 1 or 2 are also necessary to ascertain a possible reason for the division between the two. In conclusion, our data show that SCM-2 is distinguishable from SCM-1 in terms of fibrinogen binding strength. We also present here that this function strongly correlates with the ability of SCM-2 *S. canis* strains to survive in canine whole blood.

## Data availability statement

The data presented in the study are deposited in the NCBI-repository, accession number OQ983403-OQ983463.

## Ethics statement

Ethical approval was not required for the study involving animals in accordance with the local legislation and institutional requirements because blood was retrieved due to excess in diagnostics procedures.

## Author contributions

TK, OG, SH, AN, IE, and MF: designed experiments. A-ML, EA, TK, OG, AN, and IE: performed the experiments. A-ML, EA, TK, OG, SH, AN, IE, KV, and MF: analyzed the data. A-ML, EA, KV, and MF: wrote the manuscript. All authors contributed to the article and approved the submitted version.

## References

[ref1] Andrews S. (2010). “FastQC” in A quality control tool for high throughput sequence data. Cambridge, United Kingdom. Available online at: http://www.bioinformatics.babraham.ac.uk/projects/fastqc/

[ref2] Bergmann S. Eichhorn I. Kohler T. P. Hammerschmidt S. Goldmann O. Rohde M.. (2017). SCM, the M protein of *Streptococcus canis* binds immunoglobulin G. Front. Cell. Infect. Microbiol. 7:80. doi: 10.3389/fcimb.2017.00080 28401063 PMC5368172

[ref3] Bergmann R. Jentsch M. C. Uhlig A. Müller U. van der Linden M. Rasmussen M.. (2019). Prominent binding of human and equine fibrinogen to *Streptococcus equi* subsp. *zooepidemicus* is mediated by specific SzM types and is a distinct phenotype of zoonotic isolates. Infect. Immun. 88:e00559. doi: 10.1128/IAI.00559-19, PMID: 31636136 PMC6921669

[ref4] Brennan-Krohn T. (2021). Infections in animals and humans caused by bacterial 'Cousins'. Washington, DC, United States: American Society for Microbiology.

[ref5] Carlsson F. Sandin C. Lindahl G. (2005). Human fibrinogen bound to *Streptococcus pyogenes* M protein inhibits complement deposition via the classical pathway. Mol. Microbiol. 56, 28–39. doi: 10.1111/j.1365-2958.2005.04527.x, PMID: 15773976

[ref6] Chaffer M. Friedman S. Saran A. Younis A. (2005). An outbreak of *Streptococcus canis* mastitis in a dairy herd in Israel. N. Z. Vet. J. 53, 261–264. doi: 10.1080/00480169.2005.36557 16044188

[ref7] Corning B. F. Murphy J. C. Fox J. G. (1991). Group G streptococcal lymphadenitis in rats. J. Clin. Microbiol. 29, 2720–2723. doi: 10.1128/jcm.29.12.2720-2723.1991, PMID: 1757539 PMC270421

[ref8] DeWinter L. M. Low D. E. Prescott J. F. (1999). Virulence of *Streptococcus canis* from canine streptococcal toxic shock syndrome and necrotizing fasciitis. Vet. Microbiol. 70, 95–110. doi: 10.1016/S0378-1135(99)00128-5, PMID: 10591501

[ref9] DeWinter L. M. Prescott J. F. (1999). Relatedness of *Streptococcus canis* from canine streptococcal toxic shock syndrome and necrotizing fasciitis. Can. J. Vet. Res. 63, 90–95. PMID: 10369564 PMC1189525

[ref10] Fukushima Y. Tsuyuki Y. Goto M. Yoshida H. Takahashi T. (2020). Novel quinolone nonsusceptible *Streptococcus canis* strains with point mutations in quinolone resistance-determining regions and their related factors. Jpn. J. Infect. Dis. 73, 242–249. doi: 10.7883/yoken.JJID.2019.392 32009056

[ref11] Fulde M. Rohde M. Hitzmann A. Preissner K. T. Nitsche-Schmitz D. P. Nerlich A.. (2011). SCM, a novel M-like protein from *Streptococcus canis*, binds (mini)-plasminogen with high affinity and facilitates bacterial transmigration. Biochem. J. 434, 523–535. doi: 10.1042/BJ20101121 21210764

[ref12] Fulde M. Rohde M. Polok A. Preissner K. T. Chhatwal G. S. Bergmann S. (2013). Cooperative plasminogen recruitment to the surface of *Streptococcus canis* via M protein and enolase enhances bacterial survival. mBio 4, e00629–e00612. doi: 10.1128/mBio.00629-12, PMID: 23481605 PMC3604778

[ref13] Fulde M. Valentin-Weigand P. (2013). Epidemiology and pathogenicity of zoonotic streptococci. Curr. Top. Microbiol. Immunol. 368, 49–81. doi: 10.1007/82_2012_277 23192319

[ref14] Galpérine T. Cazorla C. Blanchard E. Boineau F. Ragnaud J. M. Neau D. (2007). *Streptococcus canis* infections in humans: retrospective study of 54 patients. J. Infect. 55, 23–26. doi: 10.1016/j.jinf.2006.12.013 17320186

[ref15] Hoang D. T. Chernomor O. von Haeseler A. Minh B. Q. Vinh L. S. (2018). UFBoot2: improving the ultrafast bootstrap approximation. Mol. Biol. Evol. 35, 518–522. doi: 10.1093/molbev/msx281, PMID: 29077904 PMC5850222

[ref16] Iglauer F. Kunstýr I. Mörstedt R. Farouq H. Wullenweber M. Damsch S. (1991). *Streptococcus canis* arthritis in a cat breeding colony. J. Exp. Anim. Sci. 34, 59–65. PMID: 1883871

[ref17] Kalyaanamoorthy S. Minh B. Q. Wong T. K. F. von Haeseler A. Jermiin L. S. (2017). ModelFinder: fast model selection for accurate phylogenetic estimates. Nat. Methods 14, 587–589. doi: 10.1038/nmeth.4285, PMID: 28481363 PMC5453245

[ref18] Laabei M. Ermert D. (2019). Catch me if you can: *Streptococcus pyogenes* complement evasion strategies. J. Innate Immun. 11, 3–12. doi: 10.1159/000492944, PMID: 30269134 PMC6738146

[ref19] Lacave G. Coutard A. Troché G. Augusto S. Pons S. Zuber B.. (2016). Endocarditis caused by *Streptococcus canis*: an emerging zoonosis? Infection 44, 111–114. doi: 10.1007/s15010-015-0809-3, PMID: 26104727

[ref20] Lam M. M. Clarridge J. E. III Young E. J. Mizuki S. (2007). The other group G *Streptococcus*: increased detection of *Streptococcus canis* ulcer infections in dog owners. J. Clin. Microbiol. 45, 2327–2329. doi: 10.1128/JCM.01765-06, PMID: 17475761 PMC1932974

[ref21] Lamm C. G. Ferguson A. C. Lehenbauer T. W. Love B. C. (2010). Streptococcal infection in dogs: a retrospective study of 393 cases. Vet. Pathol. 47, 387–395. doi: 10.1177/0300985809359601 20382824

[ref22] Lämmler C. Frede C. Gürtürk K. Hildebrand A. Blobel H. (1988). Binding activity of *Streptococcus canis* for albumin and other plasma proteins. J. Gen. Microbiol. 134, 2317–2323. doi: 10.1099/00221287-134-8-2317, PMID: 3253409

[ref23] Letunic I. Bork P. (2021). Interactive tree of life (iTOL) v5: an online tool for phylogenetic tree display and annotation. Nucleic Acids Res. 49, W293–w296. doi: 10.1093/nar/gkab301, PMID: 33885785 PMC8265157

[ref24] Maguin E. Duwat P. Hege T. Ehrlich D. Gruss A. (1992). New thermosensitive plasmid for gram-positive bacteria. J. Bacteriol. 174, 5633–5638. doi: 10.1128/jb.174.17.5633-5638.1992, PMID: 1324906 PMC206509

[ref25] Miller C. W. Prescott J. F. Mathews K. A. Betschel S. D. Yager J. A. Guru V.. (1996). Streptococcal toxic shock syndrome in dogs. J. Am. Vet. Med. Assoc. 209, 1421–1426. PMID: 8870738

[ref26] Minh B. Q. Schmidt H. A. Chernomor O. Schrempf D. Woodhams M. D. von Haeseler A.. (2020). IQ-TREE 2: new models and efficient methods for phylogenetic inference in the genomic era. Mol. Biol. Evol. 37, 1530–1534. doi: 10.1093/molbev/msaa015 32011700 PMC7182206

[ref27] Nerlich A. Lapschies A. M. Kohler T. P. Cornax I. Eichhorn I. Goldmann O.. (2019). Homophilic protein interactions facilitate bacterial aggregation and IgG-dependent complex formation by the *Streptococcus canis* M protein SCM. Virulence 10, 194–206. doi: 10.1080/21505594.2019.1589362, PMID: 30829556 PMC6527014

[ref28] Nikolaisen N. K. Lassen D. C. K. Chriél M. Larsen G. Jensen V. F. Pedersen K. (2017). Antimicrobial resistance among pathogenic bacteria from mink (*Neovison vison*) in Denmark. Acta Vet. Scand. 59:60. doi: 10.1186/s13028-017-0328-6, PMID: 28903765 PMC5598060

[ref29] Ohtaki H. Ohta H. Miyazaki T. Yonetamari J. Ito H. Seishima M.. (2013). A case of sepsis caused by *Streptococcus canis* in a dog owner: a first case report of sepsis without dog bite in Japan. J. Infect. Chemother. 19, 1206–1209. doi: 10.1007/s10156-013-0625-6, PMID: 23740090

[ref30] Pinho M. D. Foster G. Pomba C. Machado M. P. Baily J. L. Kuiken T.. (2019). *Streptococcus canis* are a single population infecting multiple animal hosts despite the diversity of the universally present M-like protein SCM. Front. Microbiol. 10:631. doi: 10.3389/fmicb.2019.00631, PMID: 30984150 PMC6450190

[ref31] Robinson J. H. Kehoe M. A. (1992). Group A streptococcal M proteins: virulence factors and protective antigens. Immunol. Today 13, 362–367. doi: 10.1016/0167-5699(92)90173-5 1281632

[ref32] Sanderson-Smith M. de Oliveira D. M. Guglielmini J. McMillan D. Vu T. Holien J. K.. (2014). A systematic and functional classification of *Streptococcus pyogenes* that serves as a new tool for molecular typing and vaccine development. J. Infect. Dis. 210, 1325–1338. doi: 10.1093/infdis/jiu260, PMID: 24799598 PMC6083926

[ref33] Schindelin J. Arganda-Carreras I. Frise E. Kaynig V. Longair M. Pietzsch T.. (2012). Fiji: an open-source platform for biological-image analysis. Nat. Methods 9, 676–682. doi: 10.1038/nmeth.2019 22743772 PMC3855844

[ref34] Sievers F. Wilm A. Dineen D. Gibson T. J. Karplus K. Li W.. (2011). Fast, scalable generation of high-quality protein multiple sequence alignments using Clustal Omega. Mol. Syst. Biol. 7:539. doi: 10.1038/msb.2011.75 21988835 PMC3261699

[ref35] Smeesters P. R. Botteaux A. (2020). The *emm*-cluster typing system. Methods Mol. Biol. 2136, 25–31. doi: 10.1007/978-1-0716-0467-0_3, PMID: 32430811

[ref36] Taniyama D. Abe Y. Sakai T. Kikuchi T. Takahashi T. (2017). Human case of bacteremia caused by *Streptococcus canis* sequence type 9 harboring the *scm* gene. IDCases 7, 48–52. doi: 10.1016/j.idcr.2017.01.002, PMID: 28180088 PMC5295620

[ref37] Timoney J. F. Velineni S. Ulrich B. Blanchard P. (2017). Biotypes and ScM types of isolates of *Streptococcus canis* from diseased and healthy cats. Vet. Rec. 180:358. doi: 10.1136/vr.103868, PMID: 28077757

[ref38] Verkühlen G. Pägelow D. Valentin-Weigand P. Fulde M. (2016). SCM-positive *Streptococcus canis* are predominant among pet-associated group G streptococci. Berl. Munch. Tierarztl. Wochenschr. 129, 247–250. doi: 10.2376/0005-9366-129-15060 27344918

[ref39] Whitnack E. Beachey E. H. (1985). Inhibition of complement-mediated opsonization and phagocytosis of *Streptococcus pyogenes* by D fragments of fibrinogen and fibrin bound to cell surface M protein. J. Exp. Med. 162, 1983–1997. doi: 10.1084/jem.162.6.1983, PMID: 3906018 PMC2187975

[ref40] Wick R. R. Judd L. M. Gorrie C. L. Holt K. E. (2017). Unicycler: resolving bacterial genome assemblies from short and long sequencing reads. PLoS Comput. Biol. 13:e1005595. doi: 10.1371/journal.pcbi.1005595 28594827 PMC5481147

[ref41] Yang J. Liu Y. Xu J. Li B. (2010). Characterization of a new protective antigen of *Streptococcus canis*. Vet. Res. Commun. 34, 413–421. doi: 10.1007/s11259-010-9414-1, PMID: 20490660

