# Supplementary material for: The type-2 Streptococcus canis M protein SCM-2 binds fibrinogen and facilitates antiphagocytic properties
Source: Front Microbiol. 2023 Oct 26;14:1228472. doi: 10.3389/fmicb.2023.1228472 (PMC10641296; doi:10.3389/fmicb.2023.1228472)
Supplement: Supplementary file 1 [file Data_Sheet_1.PDF]

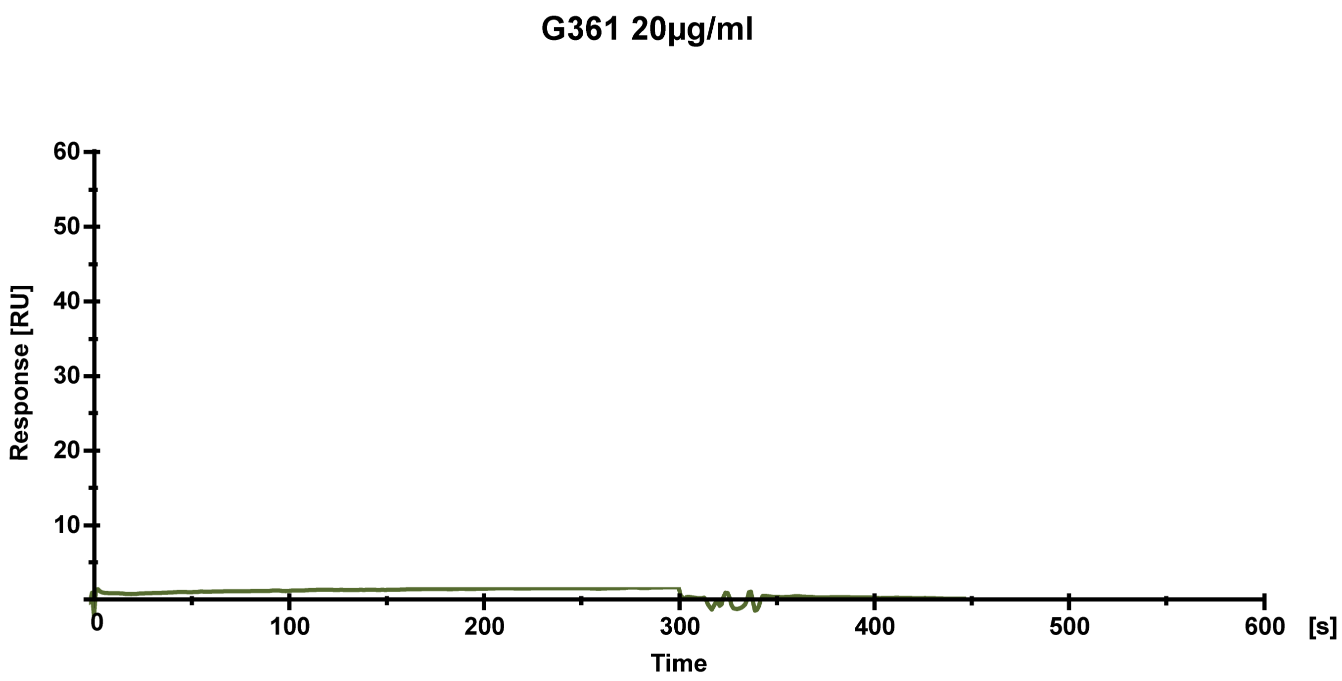

Figure S1: Analysis of the fibrinogen binding capacitiy of SCM-1 expressing *S. canis* reference strain G361 (negative control). Interactions of soluble SCM with immobilized fibrinogen was analyzed by surface plasmon resonance spectroscopy (SPR). Recombinant SCM-1 was purified as described earlier [23]. Association and dissociation of SCM-1 with immobilized fibrinogen was monitored.

A

Quality Control | Report | Residuals | Parameters

Curve	ka (1/Ms)	kd (1/s)	KD (M)	Rmax (RU)	Conc (M)	tc	Flow (ul/min)	kt (RU/Ms)	RI (RU)	Chi^2 (RU^2)	U-value
	6,368E+4	9,891E-4	1,553E-8	46,56		2,115E+21				4,15	3
Cycle: 5 1,25 µg/ml					3,125E-8		10,00	4,558E+21	4,328		
Cycle: 6 2,5 µg/ml					6,250E-8		10,00	4,558E+21	3,628		
Cycle: 7 5 µg/ml					1,250E-7		10,00	4,558E+21	2,019		
Cycle: 9 10 µg/ml					2,500E-7		10,00	4,558E+21	2,937		
Cycle: 10 20 µg/ml					5,000E-7		10,00	4,558E+21	5,792		

Quality Control | Report | Residuals | Parameters

Curve	ka (1/Ms)	kd (1/s)	KD (M)	Rmax (RU)	Conc (M)	tc	Flow (ul/min)	kt (RU/Ms)	RI (RU)	Chi^2 (RU^2)	U-value
	6,270E+4	9,482E-4	1,512E-8	46,36		5,659E+14				3,98	3
Cycle: 5 1,25 µg/ml					3,125E-8		10,00	1,219E+15	3,982		
Cycle: 6 2,5 µg/ml					6,250E-8		10,00	1,219E+15	3,433		
Cycle: 7 5 µg/ml					1,250E-7		10,00	1,219E+15	1,897		
Cycle: 9 10 µg/ml					2,500E-7		10,00	1,219E+15	1,896		
Cycle: 10 20 µg/ml					5,000E-7		10,00	1,219E+15	5,189		

Quality Control | Report | Residuals | Parameters

Curve	ka (1/Ms)	kd (1/s)	KD (M)	Rmax (RU)	Conc (M)	tc	Flow (ul/min)	kt (RU/Ms)	RI (RU)	Chi^2 (RU^2)	U-value
	6,454E+4	9,223E-4	1,429E-8	47,23		3,184E+21				4,58	4
Cycle: 5 1,25 µg/ml					3,125E-8		10,00	6,859E+21	4,418		
Cycle: 6 2,5 µg/ml					6,250E-8		10,00	6,859E+21	3,437		
Cycle: 7 5 µg/ml					1,250E-7		10,00	6,859E+21	1,675		
Cycle: 9 10 µg/ml					2,500E-7		10,00	6,859E+21	1,842		
Cycle: 10 20 µg/ml					5,000E-7		10,00	6,859E+21	5,545		

B

Quality Control | Report | Residuals | Parameters

Curve	ka (1/Ms)	kd (1/s)	KD (M)	Rmax (RU)	Conc (M)	tc	Flow (ul/min)	kt (RU/Ms)	RI (RU)	Chi^2 (RU^2)	U-value
	1,220E+4	0,002157	1,768E-7	57,09		3,426E+20				2,07	2
Cycle: 5 1,25 µg/ml					3,125E-8		10,00	7,380E+20	-0,1718		
Cycle: 6 2,5 µg/ml					6,250E-8		10,00	7,380E+20	0,9667		
Cycle: 7 5 µg/ml					1,250E-7		10,00	7,380E+20	3,037		
Cycle: 9 10 µg/ml					2,500E-7		10,00	7,380E+20	6,305		
Cycle: 10 20 µg/ml					5,000E-7		10,00	7,380E+20	12,29		

Quality Control | Report | Residuals | Parameters

Curve	ka (1/Ms)	kd (1/s)	KD (M)	Rmax (RU)	Conc (M)	tc	Flow (ul/min)	kt (RU/Ms)	RI (RU)	Chi^2 (RU^2)	U-value
	1,276E+4	0,002397	1,878E-7	52,78		7,734E+15				1,99	2
Cycle: 5 1,25 µg/ml					3,125E-8		10,00	1,666E+16	0,03562		
Cycle: 6 2,5 µg/ml					6,250E-8		10,00	1,666E+16	1,298		
Cycle: 7 5 µg/ml					1,250E-7		10,00	1,666E+16	3,029		
Cycle: 9 10 µg/ml					2,500E-7		10,00	1,666E+16	6,114		
Cycle: 10 20 µg/ml					5,000E-7		10,00	1,666E+16	12,08		

Quality Control | Report | Residuals | Parameters

Curve	ka (1/Ms)	kd (1/s)	KD (M)	Rmax (RU)	Conc (M)	tc	Flow (ul/min)	kt (RU/Ms)	RI (RU)	Chi^2 (RU^2)	U-value
	1,306E+4	0,002714	2,079E-7	46,72		4,898E+15				1,81	2
Cycle: 5 1,25 µg/ml					3,125E-8		10,00	1,055E+16	0,2222		
Cycle: 6 2,5 µg/ml					6,250E-8		10,00	1,055E+16	1,307		
Cycle: 7 5 µg/ml					1,250E-7		10,00	1,055E+16	2,805		
Cycle: 9 10 µg/ml					2,500E-7		10,00	1,055E+16	5,425		
Cycle: 10 20 µg/ml					5,000E-7		10,00	1,055E+16	12,10		

Figure S2: Supporting information on surface plasmon resonance spectroscopy (SPR) for SCM-2 expressing *S. canis* strains IMT40096 (A) and IMT42870 (B). Results from SPR corresponding to association and dissociation of SCM-2 and immoblizied fibrinogen are graphically illustrated in **Fig. 4**.

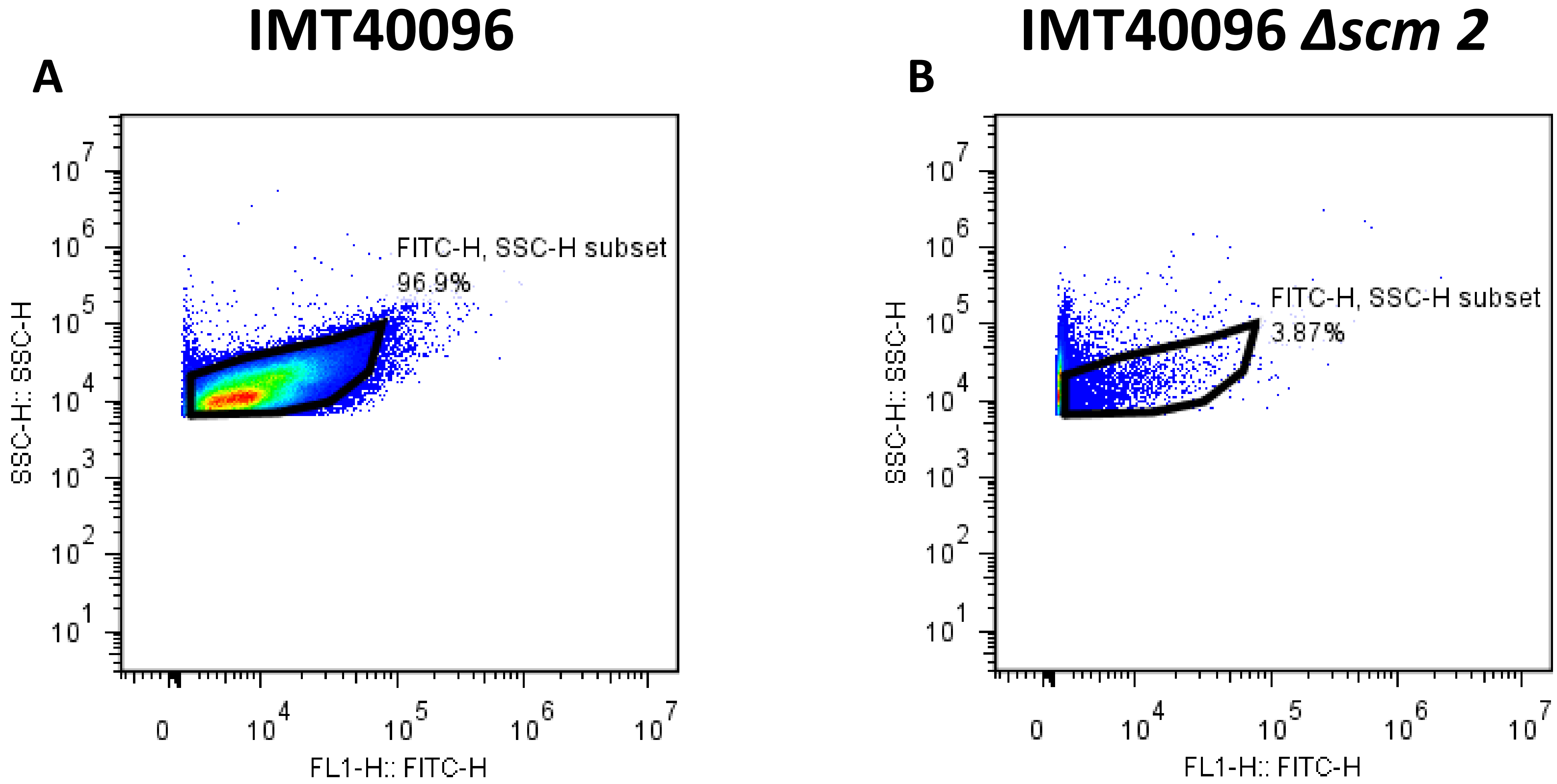

Fig. S3: Gating strategy of FACS analysis applied to all *S.canis* samples tested for fibrinogen binding. 10^4 cells were gated on Side scatter area (SSC-H) vs Fluorescin isothiocyanate labeled human fibrinogen from Molecular Innovations (FITC-H). (A) IMT40096 served as positive control and was used as gating reference for the succeeding samples. (B) negative control containing the *Δscm2* mutant.
