# Supplementary material for: The type-2 Streptococcus canis M protein SCM-2 binds fibrinogen and facilitates antiphagocytic properties
Source: Front Microbiol. 2023 Oct 26;14:1228472. doi: 10.3389/fmicb.2023.1228472 (PMC10641296; doi:10.3389/fmicb.2023.1228472)
Supplement: Supplementary file 2 [file Data_Sheet_2.PDF]

Supplementary table 1: Metadata of *S. canis* isolates used in this study

Strain	Host	Collection date	Tissue type	City	Country	Accession number	SCM classification
AGF788	Human	13.03.2019	Midstream urine	Hannover	Germany	OQ983403	SCM 2
AGF793	Human	13.03.2019	Wound	Berlin	Germany	OQ983404	SCM 1
AGF795	Human	13.03.2019	Wound	Berlin	Germany	OQ983405	SCM 1
AGF801	Cow	24.04.2019	Milk	Wunstorf	Germany	OQ983406	SCM 2
AGF802	Cow	25.04.2019	Milk	Wunstorf	Germany	OQ983407	SCM 1
AGF804	Cow	26.04.2019	Milk	Wunstorf	Germany	OQ983408	SCM 2
AGF871	Human	14.06.2019	Vaginal	Aachen	Germany	OQ983409	SCM 1
AGF872	Human	14.06.2019	Blood	Aachen	Germany	OQ983410	SCM 1
AGF873	Human	14.06.2019	Unknown	Aachen	Germany	OQ983411	SCM 1
AGF874	Human	14.06.2019	Blood	Aachen	Germany	OQ983412	SCM 1
AGF875	Human	15.06.2019	Vaginal	Aachen	Germany	OQ983413	SCM 2
AGF876	Human	16.06.2019	Vaginal	Aachen	Germany	OQ983414	SCM 1
AGF877	Human	17.06.2019	Blood	Aachen	Germany	OQ983415	SCM 2
AGF878	Human	18.06.2019	Wound	Aachen	Germany	OQ983416	SCM 1
AGF914	Mustelid	29.07.2019	Unknown	Lyngby	Denmark	OQ983417	SCM 1
AGF915	Mustelid	29.07.2019	Unknown	Lyngby	Denmark	OQ983418	SCM 2
AGF916	Mustelid	29.07.2019	Unknown	Lyngby	Denmark	OQ983419	SCM 1
AGF917	Mustelid	29.07.2019	Unknown	Lyngby	Denmark	OQ983420	SCM 1
AGF918	Mustelid	29.07.2019	Unknown	Lyngby	Denmark	OQ983421	SCM 2
AGF919	Mustelid	29.07.2019	Unknown	Lyngby	Denmark	OQ983422	SCM 1
AGF920	Mustelid	29.07.2019	Unknown	Lyngby	Denmark	OQ983423	SCM 1
AGF921	Mustelid	29.07.2019	Unknown	Lyngby	Denmark	OQ983424	SCM 1
IMT39418	Dog	11.05.2016	Otitis Externa	Berlin	Germany	OQ983425	SCM 1
IMT39430	Dog	30.05.2016	Unknown	Berlin	Germany	OQ983426	SCM 2
IMT39470	Dog	01.12.2016	Abscess	Berlin	Germany	OQ983427	SCM 1
IMT39845	Dog	15.08.2016	Otitis Externa	Berlin	Germany	OQ983428	SCM 1
IMT40096	Cat	05.12.2016	Peritonitis	Buer	Germany	OQ983429	SCM 2
IMT40100	Cat	14.10.2016	Feline Viral Rhinotracheitis	Berlin	Germany	OQ983430	SCM 1
IMT40165	Dog	21.04.2016	Unknown	Berlin	Germany	OQ983431	SCM 1
IMT40238	Cat	01.12.2016	Feline Viral Rhinotracheitis	Berlin	Germany	OQ983432	SCM 1
IMT40455	Dog	25.11.2016	Pyodermia	Düsseldorf	Germany	OQ983433	SCM 2
IMT40614	Dog	08.12.2016	Otitis Externa	Berlin	Germany	OQ983434	SCM 2
IMT40791	Dog	19.12.2016	Otitis Externa	Premnitz	Germany	OQ983435	SCM 1
IMT40792	Dog	20.12.2016	Unknown	Berlin	Germany	OQ983436	SCM 1
IMT40795	Dog	20.12.2016	Abscess	Berlin	Germany	OQ983437	SCM 1
IMT40953	Dog	18.01.2017	Unknown	Gelsenkirchen	Germany	OQ983438	SCM 1
IMT41389	Dog	19.01.2017	Pyodermia	Berlin	Germany	OQ983439	SCM 2
IMT41390	Dog	19.01.2017	Joint puncture	Berlin	Germany	OQ983440	SCM 1
IMT41466	Dog	10.02.2017	Necrosis	Berlin	Germany	OQ983441	SCM 1
IMT41564	Dog	21.02.2017	Otitis Externa	Berlin	Germany	OQ983442	SCM 1
IMT41640	Dog	24.02.2017	Death	Berlin	Germany	OQ983443	SCM 1
IMT41746	Dog	06.03.2017	Polydipsia	Berlin	Germany	OQ983444	SCM 2
IMT41753	Dog	16.02.2017	Unknown	Zossen	Germany	OQ983445	SCM 1
IMT42047	Cat	11.04.2017	Azotemia	Berlin	Germany	OQ983446	SCM 2
IMT42071	Dog	21.04.2017	Unknown	Berlin	Germany	OQ983447	SCM 1
IMT42100	Dog	21.04.2017	Unknown	Berlin	Germany	OQ983448	SCM 1
IMT42201	Dog	04.05.2017	Myelopathia	Berlin	Germany	OQ983449	SCM 1
IMT42202	Dog	04.05.2017	Myelopathia	Lindchen	Germany	OQ983450	SCM 1
IMT42204	Dog	04.05.2017	Otitis Externa	Berlin	Germany	OQ983451	SCM 1
IMT42253	Dog	09.05.2017	Otitis Externa	Diedersdorf	Germany	OQ983452	SCM 1
IMT42340	Dog	15.05.2017	Pneumonia	Berlin	Germany	OQ983453	SCM 1
IMT42341	Dog	15.05.2017	Otitis Externa	Berlin	Germany	OQ983454	SCM 1
IMT42382	Dog	24.05.2017	Arthritis	Stahndorf	Germany	OQ983455	SCM 1
IMT42859	Dog	16.06.2017	Otitis Externa	Solingen	Germany	OQ983456	SCM 1
IMT42862	Dog	16.06.2017	Otitis Externa	Berlin	Germany	OQ983457	SCM 1
IMT42867	Dog	16.06.2017	Otitis Externa	Berlin	Germany	OQ983458	SCM 1
IMT42870	Dog	19.06.2017	Fever	Berlin	Germany	OQ983459	SCM 2
IMT43233	Dog	06.07.2017	Otitis Externa	Berlin	Germany	OQ983460	SCM 1
IMT43373	Dog	25.07.2017	Arthritis	Berlin	Germany	OQ983461	SCM 1
S.canis 322	Unknown	Unknown	Unknown	Unknown	Unknown	OQ983462	SCM 2
S.canis G2	Unknown	Unknown	Unknown	Unknown	Unknown	OQ983463	SCM 2
S.canis G361	Human	01.05.2006	Vaginal Swab	Hannover	Germany	NMRV00000000	SCM 1
S.canis SRUC003	Dog	1996	Prepuce	Unknown	Scotland	MH996675	SCM 2
S.canis SRUC005	Dog	1996	Ear Exudate	Unknown	Scotland	MH996677	SCM 2
S.canis SRUC034	Dog	1998	Interdigital cyst	Unknown	Scotland	MH996678	SCM 2
S.canis SRUC036	Dog	1998	Urine	Unknown	Scotland	MH996667	SCM 2
S.canis SRUC056	Dog	1999	Ear Exudate	Unknown	Scotland	MH996679	SCM 2
S.canis SRUC072	Dog	2007	Vaginal Exudate	Unknown	Scotland	MH996676	SCM 2
S.canis SRUC095	Badger	1993	Unknown	Unknown	Scotland	MH996672	SCM 2
S.canis SRUC096	Badger	1996	Unknown	Unknown	Scotland	MH996673	SCM 2
S.canis SRUC097	Fox	2014	Pleural Fluid	Unknown	Scotland	MH996668	SCM 2
S.canis FMV1451.06	Dog	2006	Ear Exudate	Unknown	Portugal	MH996671	SCM 2
S.canis FMV2322.02	Dog	2002	Ear Exudate	Unknown	Portugal	MH996669	SCM 2
S.canis FMV3662.06	Dog	2006	Ear Exudate	Unknown	Portugal	MH996674	SCM 2
S.canis FMVES5.02	Dog	2002	Skin Exudate	unknown	Portugal	MH996670	SCM 2
